# Preliminary Study of New Gallium-68 Radiolabeled Peptide Targeting NRP-1 to Detect Brain Metastases by Positron Emission Tomography

**DOI:** 10.3390/molecules26237273

**Published:** 2021-11-30

**Authors:** Albert Moussaron, Valérie Jouan-Hureaux, Charlotte Collet, Julien Pierson, Noémie Thomas, Laurence Choulier, Nicolas Veran, Matthieu Doyen, Philippe Arnoux, Fatiha Maskali, Dominique Dumas, Samir Acherar, Muriel Barberi-Heyob, Céline Frochot

**Affiliations:** 1 Université de Lorraine, CNRS, LRGP, F-54000 Nancy, France; albert.moussaron@hotmail.fr (A.M.); philippe.arnoux@univ-lorraine.fr (P.A.); 2 Université de Lorraine, CNRS, CRAN, F-54000 Nancy, France; valerie.jouan-hureaux@univ-lorraine.fr (V.J.-H.); julien.pierson@univ-lorraine.fr (J.P.); noemie.thomas@univ-lorraine.fr (N.T.); muriel.barberi-heyob@univ-lorraine.fr (M.B.-H.); 3 Nancyclotep Molecular Imaging Platform, F-54500 Vandœuvre-lès-Nancy, France; charlotte.collet@univ-lorraine.fr (C.C.); n.veran@chru-nancy.fr (N.V.); m.doyen@nancyclotep.com (M.D.); f.maskali@nancyclotep.com (F.M.); 4 Université de Lorraine, INSERM, U1254, IADI, F-54500 Vandœuvre-lès-Nancy, France; 5 Université de Strasbourg, CNRS, LBP, F-67000 Strasbourg, France; laurence.choulier@unistra.fr; 6 Université de Lorraine, CNRS, IMoPA, F-54000 Nancy, France; dominique.dumas@univ-lorraine.fr; 7 Université de Lorraine, CNRS, LCPM, F-54000 Nancy, France; samir.acherar@univ-lorraine.fr

**Keywords:** radiolabeling, brain metastases, fluorescence, peptide, NRP-1, targeting

## Abstract

Due to their very poor prognosis and a fatal outcome, secondary brain tumors are one of the biggest challenges in oncology today. From the point of view of the early diagnosis of these brain micro- and macro-tumors, the sensitivity and specificity of the diagnostic tools constitute an obstacle. Molecular imaging, such as Positron Emission Tomography (PET), is a promising technique but remains limited in the search for cerebral localizations, given the commercially available radiotracers. Indeed, the [^18^F]FDG PET remains constrained by the physiological fixation of the cerebral cortex, which hinders the visualization of cerebral metastases. Tumor angiogenesis is recognized as a crucial phenomenon in the progression of malignant tumors and is correlated with overexpression of the neuropilin-1 (NRP-1) receptor. Here, we describe the synthesis and the photophysical properties of the new gallium-68 radiolabeled peptide to target NRP-1. The KDKPPR peptide was coupled with gallium-68 anchored into a bifunctional NODAGA chelating agent, as well as Cy5 for fluorescence detection. The Cy5 absorbance spectra did not change, whereas the molar extinction coefficient (ε) decreased drastically. An enhancement of the fluorescence quantum yield (φ_F_) could be observed due to the better water solubility of Cy5. [^68^Ga]Ga-NODAGA-K(Cy5)DKPPR was radiosynthesized efficiently, presented hydrophilic properties (log D = −1.86), and had high in vitro stability (>120 min). The molecular affinity and the cytotoxicity of this new chelated radiotracer were evaluated in vitro on endothelial cells (HUVEC) and MDA-MB-231 cancer cells (hormone-independent and triple-negative line) and in vivo on a brain model of metastasis in a nude rat using the MDA-MB-231 cell line. No in vitro toxicity has been observed. The in vivo preliminary experiments showed promising results, with a high contrast between the healthy brain and metastatic foci for [^68^Ga]Ga-NODAGA-K(Cy5)DKPPR.

## 1. Introduction

From the perspective of the early diagnosis of tumors, the sensitivity and specificity of the diagnostic tools remain important challenges. Molecular imaging such as Positron Emission Tomography (PET) is a reference technique for the diagnosis of many diseases [1]. PET is a technique that is beginning to be widely used in several pathologies, such as neurology, cardiology, and inflammation. Nevertheless, it remains the most prescribed exam in nuclear oncology, with [^18^F]FDG as the gold standard. This imaging requires the use of radiotracers to locate and quantify specific targets or pathological processes. Although, it has been established that [^18^F]FDG PET has a lower sensitivity [2] in determining the presence, location, and number of metastases than MRI, especially for the detection of small lesions [3]. This suggests that PET imaging can provide valuable complementary information in the evaluation of brain tumors (aggressiveness, differentiation, etc.). [^18^F]FDG shows glucidic metabolism of the tumor and is not specific. For a few years, new radiotracers based on amino acids ([^18^F]F-DOPA, [^18^F]FET, and [^11^C]-methionine) have shown better results compared to [^18^F]FDG, but they are also not very specific [4].

The use of radiolabeled bioactive macromolecules in imaging has emerged as an important field for almost four decades in nuclear medicine [5,6]. In particular, labeled peptides or proteins are useful for PET [7,8] or preclinical and clinical research as new diagnostic agents and are today considered as fully “druggable” entities [9,10]. Research in nuclear medicine technology and the development of new radiotracers has considerably changed these last years by using more specific radiotracers. To have efficiently labeled peptides for PET applications, isotope half-lives need to be compatible with the peptide biodistribution time, and radiolabeling conditions need to be compatible with the peptide stability. Gallium-68 is a well-adapted isotope thanks to its quick synthesis times (~15 min) and easy radiochemistry (radiochemical yield >95%) by complexation into chelating groups. Numerous gallium-68 radiotracers are described in the literature; they can be designed with monomeric or polymeric [11] targeting peptides for a higher specificity.

In a search for greater specificity and due to the presence of peptide receptors overexpressed on tumor cells, peptides are the ligands of choice to develop targeted molecular imaging agents [12]. Recently, new peptide-based radiotracers have shown very promising results, such as the [^68^Ga]Ga-DOTATOC radiotracer used for clinical diagnosis applications, which is composed of a DOTA as the bifunctional agent, a peptide octreotide analog as the ligand able to target the somatostatin receptor [13]. In the past decade, the work of some researchers in Nancy has led to the development of targeting strategies via peptides to treat brain tumor glioblastoma [14]. Tumor angiogenesis is recognized as a crucial phenomenon in the progression of malignant tumors. Vascular endothelial growth factor (VEGF) is one of the most specific and important growth factors involved in the mechanisms of tumor angiogenesis. A recent evaluation has shown that angiogenesis was correlated with overexpression of the neuropilin-1 (NRP-1) receptor [15]. NRP-1 is also expressed in tumor angiogenic vessels and some tumor cells [16] and seems to be a target of choice for tumor imaging.

In vivo, the relevance of a peptide targeting NRP-1 overexpressed by angiogenic endothelial cells to detect the peripheral infiltrative part of the high-grade brain tumor has been clearly demonstrated; over the past ten years, we have demonstrated the interest of vascular targeting via ATWLPPR, a ligand peptide of NRP-1 (recognition domain b1b2), but also its instability in vivo [17,18,19,20]. The work of Wu et al. [21] also demonstrated the interest of targeting NRP-1 with the ATWLPPR peptide coupled to Fluor-18. Recently, we developed and tested other peptides that had more affinity for NRP-1, such as DKPPR [22,23]. In this study, the KDKPPR peptide sequence was chosen to be coupled to a bifunctional chelator and a fluorescent agent. We have already demonstrated that the coupling of a porphyrin [24] or gold nanoparticles [14] to this sequence does not modify its affinity for NRP-1.

In this paper, we describe the synthesis and the photophysical properties of the new gallium-68 radiolabeled KDKPPR peptide to target NRP-1. KDKPPR has been slightly modified, allowing labeling with gallium-68 anchored into a bifunctional NODAGA chelating agent, as well as Cy5 fluorophore for fluorescence detection. The cytotoxicity and the molecular affinity of this new chelated radiotracer have been evaluated in vitro and in vivo on a brain model of metastasis in a nude rat using MDA-MB-231 cells (hormone-independent and triple-negative line). For this purpose, we chose two in vitro models: HUVEC (angiogenic endothelial cell model) for the targeting of the tumor vascular network and MDA-MB-231 (triple-negative breast cancer cell line) for the targeting of small metastatic sites. In vivo experiments were conducted using a nude rat model of brain metastasis with an intracarotid injection of MDA-MB-231 cells.

## 2. Results

### 2.1. Synthesis

The syntheses of tracers containing a targeting peptide and a chelator, suitable for PET imaging, have already been described in the literature [[25]]. In this paper, in order to maintain the affinity of DKPPR for NRP-1 [[23,24]], a new strategy is proposed. The conjugation of the KDKPPR peptide moiety to a fluorescent probe, Cy5, is followed by the grafting of this new fluorescent probe–peptide conjugate to the bifunctional chelator, 1,4,7-triazacyclononane,1-glutaric acid-4,7-acetic acid (NODAGA), which is known to form a stable complex with Ga^3+^ [25] to produce ^nat^Ga-NODAGA-K(Cy5)DKPPR. The same synthetic route was used with a scramble peptide of DKPPR (^nat^Ga-NODAGA-K(Cy5)RPKPD, named also ^nat^Ga-NODAGA-K(Cy5)scramble) to check the targeting selectivity of this new labeled compound.

**Figure 1:**
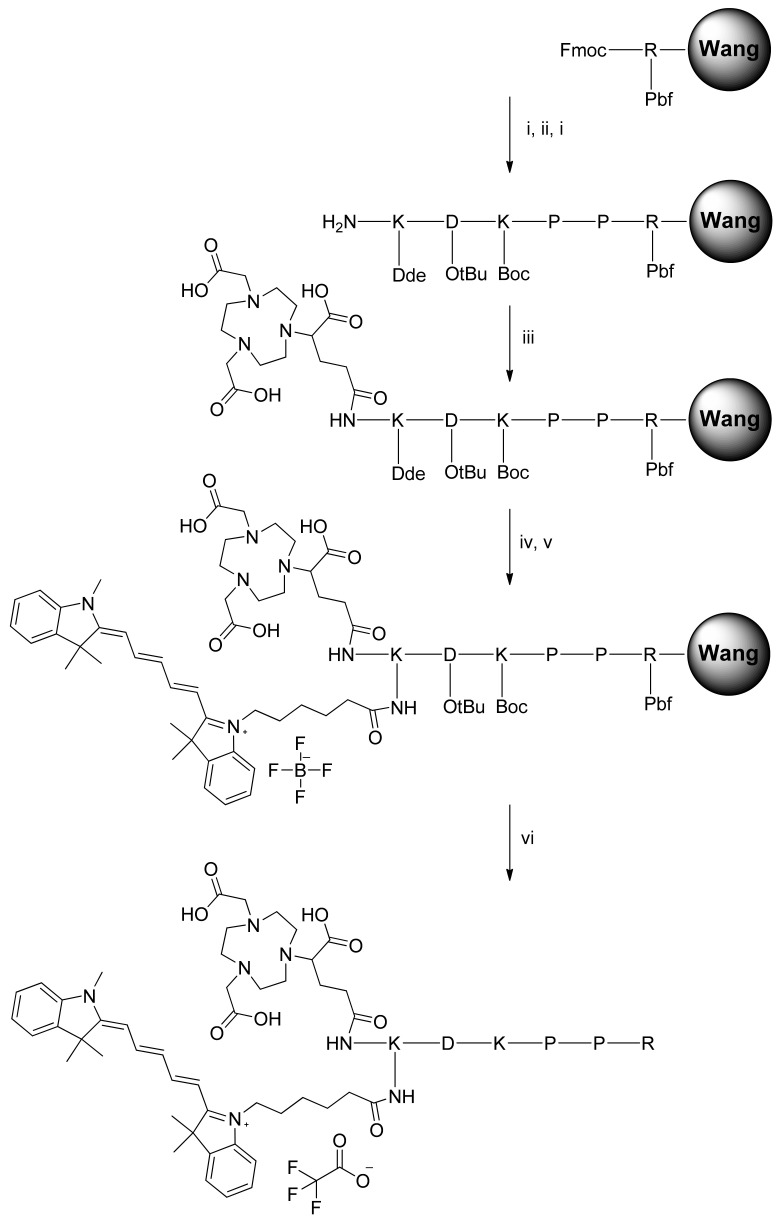
Synthesis of NODAGA-K(Cy5)DKPPR. Automatic and manual peptide synthesis: (i) Piperidine 20% in DMF, 2 × 15 min. (ii) Fmoc-PP-OH, HBTU, NMM, NMP, DMF, r.t., 2 h. (iii) NODAGA-NHS ester, DIPEA, DMF, r.t., overnight. (iv) Hydrazine monohydrate 3% in DMF, 3 × 3 min. (v) Cyanine5-NHS ester, DIPEA, DMF, r.t., overnight. (vi) TFA/TIPS/H_2_O: 92.5/2.5/5, r.t., 2 h.

The NODAGA-K(Cy5)DKPPR and NODAGA-K(Cy5)scramble compounds were synthesized by the Fmoc/tBu/Dde solid-phase strategy (Figure 1). The lysine side chain provided a convenient handle to link the fluorescent probe. After the coupling of Fmoc-Lys(Dde)-OH on the DKPPR-Wang resin and removal of the *N*-terminal Fmoc group with piperidine, the NODAGA-NHS ester was coupled. Finally, the Dde group on the Lys side was removed with hydrazine, and then, the resultant free amine was coupled with the Cy5-NHS ester. Finally, deprotection of the side chains and cleavage from the resin were then performed. The same strategy was used with the scramble peptide.

For the synthesis of K(Cy5)DKPPR and K(Cy5)scramble, we linked first Cy5-NHS in the liquid phase at the ε-NH_2_ position of Fmoc-Lys-OH, followed by the coupling of the Fmoc-Lys(Cy5)-OH to DKPPR- and scramble-Wang resins. Following cleavage from the resin and side chain deprotection using TFA/TIPS/H_2_O (95/2.5/2.5), K(Cy5)DKPPR and K(Cy5)scramble were purified by HPLC and obtained as blue powders in overall yields of 16.6% and 15%, respectively.

**Figure 2:**
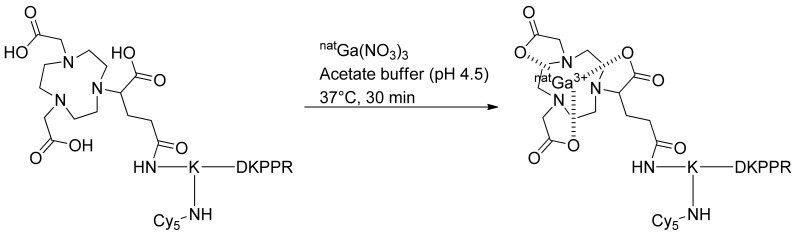
Complexation of NODAGA-K(Cy5)DKPPR with nonradioactive gallium-69.

For complexation with nonradioactive gallium-69 (^nat^Ga), NODAGA-K(Cy5)DKPPR or NODAGA-K(Cy5)scramble was incubated for 30 min with ^nat^Ga(NO_3_)_3_ in an ammonium acetate solution (pH 4.5) at 37 °C. The nonradioactive chelates were directly purified to remove the excess of ^nat^Ga, and the labeled compounds were used for the in vitro assay (Figure 2).

### 2.2. Photophysical Properties

**Figure 3:**
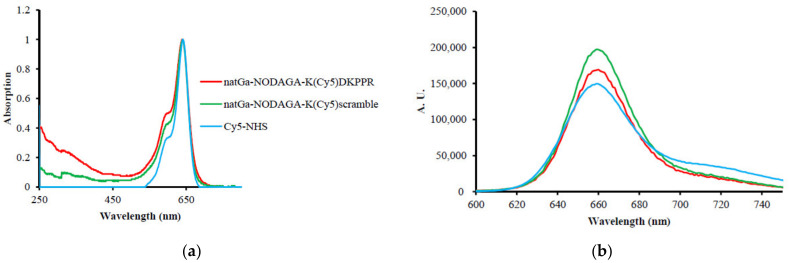
(**a**) Normalized absorption spectra in water. (**b**) Normalized fluorescence emission spectra in water (λ_ex_ = 590 nm).

No modification of the Cy5-NHS absorbance spectrum was detected after its coupling to the peptide (or scramble) and NODAGA. However, ε decreased drastically. The fluorescence quantum yield (φ_F_) of Cy5-NHS in water was 27%. After its coupling to the peptide (or scramble) and NODAGA, an enhancement of φ_F_ could be observed up to 61% for ^nat^Ga-NODAGA-K(Cy5)DKPPR and 79% for ^nat^Ga-NODAGA-K(Cy5)scramble, highlighting the fact that the NODAGA peptide improved the water solubility of Cy5 (Figure 3, Table 1).

### 2.3. Radiochemistry

**Figure 4:**
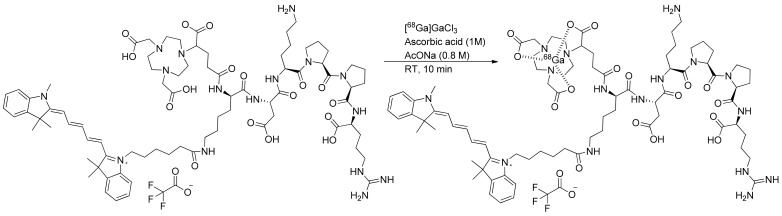
Radiosynthesis of [^68^Ga]Ga-NODAGA-K(Cy5)DKPPR.

The investigation of gallium-68 incorporation into the NODAGA chelator was screened by analyzing the effects of different amounts of precursors, reaction times, and temperatures. The radiochemical yield was precursor-dependent at 8, 15, 30, and 50 µg. This investigation revealed the efficient coordination of gallium-68 at room temperature within 10 min. In the hardest conditions (110 °C, 15 min), degradation of the compound was observed and an apparition of impurities. In optimal conditions (50 µg of NODAGA-K(Cy5)DKPPR precursor with 10 µL of ascorbic acid 1 M in 1 mL of 0.8-M AcONa at room temperature within 10 min), the incorporated yield of gallium-68 to produce [^68^Ga]Ga-NODAGA-K(Cy5)DKPPR was 94.9 ± 1.3%. Automated [^68^Ga]Ga-NODAGA-K(Cy5)DKPPR radiosynthesis on mAIO with ^68^Ga under these best conditions allowed producing [^68^Ga]Ga-NODAGA-K(Cy5)DKPPR with a 90.3 ± 2.5% (*n* = 12) decay-corrected radiochemical yield in 30 min, including the purification and formulation steps (Figure 4).

The radiochemical purity of [^68^Ga]Ga-NODAGA-K(Cy5)DKPPR (Rt = 9.0 min, corresponding with a nonradioactive reference (Figures S1 and S2) was more than 95%, and the molar activity was an average of *Am* = 6.3 ± 1.2 MBq/nmol calculated from the ratio between the total radioactivity of [^68^Ga]Ga-NODAGA-K(Cy5)DKPPR and the molar amount of NODAGA-K(Cy5)DKPPR in each preparation.

[^68^Ga]Ga-NODAGA-K(Cy5)DKPPR demonstrated a high in vitro stability remaining stable (>95%) over a period of 2 h in the final product media and in the plasma (Figure S3). The distribution coefficient (log D value) of 1.86 +/− 0.1 demonstrated hydrophilic behavior for [^68^Ga]Ga-NODAGA-K(Cy5)DKPPR.

### 2.4. Binding to Recombinant NRP-1 Protein

We evaluated the binding capacity of compounds toward NRP-1 using two different techniques: (1) a competitive binding assay with biotinylated VEGF (b-VEGF-165), a natural ligand of NRP-1 immobilized on a microplate surface in the presence of heparin, which allowed calculating the IC_50_ (the concentration of compound that displaced 50% of b-VEGF-165), and (2) surface plasmon resonance (SPR), a sensitive, optical-based, label-free detection technology for the real-time monitoring of molecular interactions between injected analytes and ligands covalently attached to the surface of a sensor chip. This technique was used to calculate the K_D_. VEGF-165 was used as the positive control in these two techniques.

**Figure 5:**
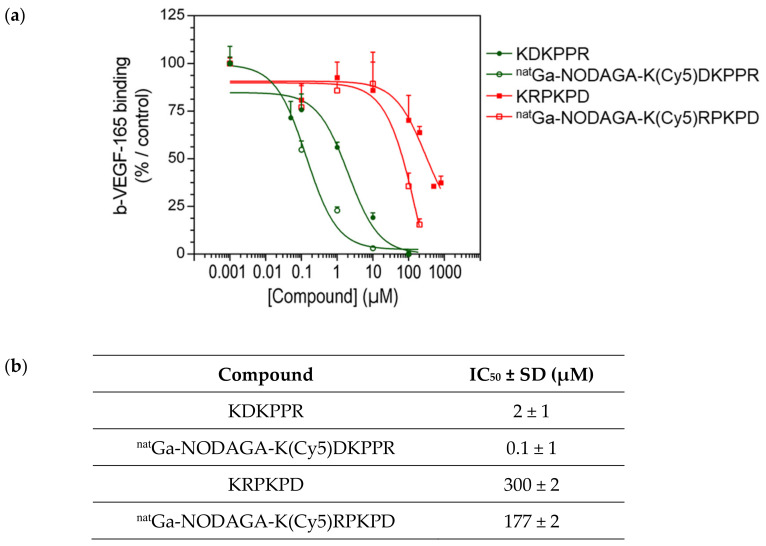
(**a**) Binding to the recombinant NRP-1 protein: competitive binding assay. Binding of b-VEGF-165 to NRP-1 was evaluated in the presence of the increasing concentration of ^nat^Ga-NODAGA-K(Cy5)DKPPR (empty green circle) or ^nat^Ga-NODAGA-K(Cy5)RPKPD (empty red square) compared with KDKPPR (circle filled with green) or KRPKPD (circle filled with red). Data points show the mean ± SD (µM), and the lines represent the one-site fit log-IC_50_ nonlinear analysis regression curve. (**b**) Binding to the recombinant NRP-1 protein: competitive binding assay. The IC_50_ were calculated using the one-site fit log-IC_50_ nonlinear analysis regression curve.

The results obtained with the binding assay showed that the KDKPPR peptide and ^nat^Ga-NODAGA-K(Cy5)DKPPR were able to displace b-VEGF-165 in a dose-dependent manner (Figure 5). VEGF-165 completely shifted the binding of b-VEGF-165 to the receptor when used at 26 nM (data not shown). Furthermore, considering the values obtained for the standard deviation, no difference was observed between KDKPPR alone and ^nat^Ga-NODAGA-K(Cy5)DKPPR, suggesting that the covalent linkage of ^nat^Ga-NODAGA and Cy5 to the KDKPPR peptide did not modify its binding onto NRP-1. Compounds with a scramble peptide (KRPKPD) or ^nat^Ga-NODAGA-K(Cy5)RPKPD) were able to decrease the binding of b-VEGF-165 to NRP-1 but only when they were used at high concentration (>100 µM). The capacity of these molecules to displace b-VEGF-165 was very weak, as indicated by the IC_50_ values (300 and 177 µM, respectively).

**Figure 6:**
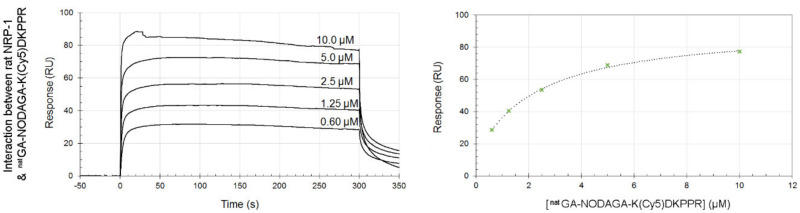
SPR experiments. Sensorgrams (**left**) and titration (**right**) curves corresponding to the interactions between rat-NRP-1 and ^nat^Ga-NODAGA-K(Cy5)DKPPR. Briefly, IgG1 and recombinant rat NRP-1 were immobilized on a CM5 sensor chip. ^nat^Ga-NODAGA-K(Cy5)DKPPR (0.60–10 µM) was injected at a flow rate of 30 µL.min^−1^ at a temperature of 25 °C. Data were recorded and presented as the response (RU) as a function of time (s) after double-referencing: subtraction of the signal obtained on the reference IgG1 surface and subtraction of the buffer. These sensorgrams (**left side**) were used to draw titration curves (**right side**) by plotting responses recorded 5 s before the end of the injection as a function of the analyte concentrations.

The results obtained by SPR technology showed affinities for NRP-1 at the micromolar level for all the compounds conjugated to the KDKPPR peptide (KDKPPR, K(Cy5)DKPPR, and ^nat^Ga-NODAGA-K(Cy5)DKPPR) (Figure 6 and Figure S4, Table 2). The K_D_ values found were of the order of micromolars and closed to those previously found for KDKPPR (8.7 µM) [25]. No statistical differences were observed between all the compounds, indicating that the conjugation of Cy5 and ^nat^Ga-NODAGA to KDKPPR did not influence its interaction towards NRP-1.The nonideal shape of the curve recorded for the compounds conjugated to KRPKPD (somewhat irregular plateau level, remaining signal at the end of the post-injection period) clearly denoted a weak affinity or a nonspecific interaction of the KRPKPD peptide, K(Cy5)RPKPD, or ^nat^Ga-NODAGA-K(Cy5)RPKPD toward NRP-1 (see Supplementary Data Figure S5). b-VEGF-165 was also used as the positive control (see Supplementary Data Figure S5).

We found a good correlation between the results obtained by SPR and the competitive binding assay, indicating that the covalent coupling of Cy5 and ^nat^Ga-NODAGA to the KDKPPR peptide did not modify its ability to interact with NRP-1. ^nat^Ga-NODAGA-K(Cy5)DKPPR is a good candidate for the diagnosis of NRP-1.

### 2.5. NRP-1 Protein Expression of Cells

The expression of NRP-1 protein in MDA-MB-231 and HUVEC cells was evaluated by a Western blotting analysis. MDA-MB-231 cells overexpress NRP-1 in comparison with HUVEC cells, in which NRP-1 was found at a lower level (Figure S6). NRP-1 expression was also observed in the in vivo metastasis model (Figure S7).

### 2.6. Metabolic Activity and Proliferation Assay

A metabolic activity assay (MTS) was realized to evaluate the cellular toxicity of the NRP-1-targeting compounds after 4 h and 24 h of exposure of MDA-MB-231 or HUVEC cells. In the range of 0–10 µM of concentration, no significant difference in cellular viability was observed whatever cell line was used and exposure time (Figure S8). The same results were obtained with the compounds coupled with the scramble.

**Figure 7:**
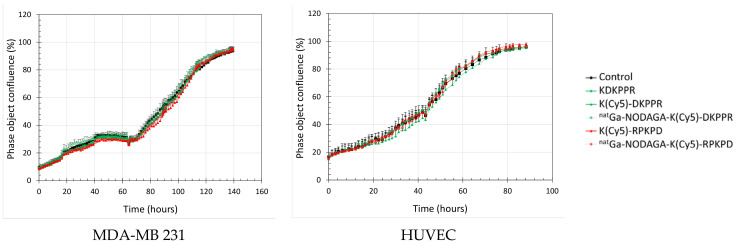
Real-time cell tracking: cell confluence as a function of time. MDA-MB-231 (**left**) or HUVEC (**right**) cells were allowed to grow before being exposed to the culture media (control) (black) or 10 µM of the NRP-1-targeting (green) or scramble (red) compounds until the end of the experiment: peptide alone (full box, solid line) and ^nat^Ga-NODAGA conjugate (empty box, dotted line).

We also evaluated the antiproliferative effect of the NRP-1-targeting compounds by the real-time follow-up of MDA-MB-231 and HUVEC cells (Figure 7). The cellular confluency was calculated by using contrast-phase photographs taken every 2 h. The cells were allowed to grow and were then exposed at the start of the exponential phase of growth to 0–10 µM of the compounds until the end of experimentation. Cells exposed to NRP-1 targeting or the scramble compounds proliferated normally, as obtained for the control. The doubling time calculated during the exponential phase of the growth was equivalent for the two cell lines (Table 3) and was not modified during the exposure times of the compounds (34 h for MDA-MB-231 and 29 h for HUVEC). The morphology of the cells was not modified by treatment (Figure S9). All of these results indicated that ^nat^Ga-NODAGA-K(Cy5)DKPPR did not modify the metabolic activity and the proliferation of cells and was thus not toxic in the range of 0–10 µM.

### 2.7. In Vivo Preliminary Analysis

**Figure 8:**
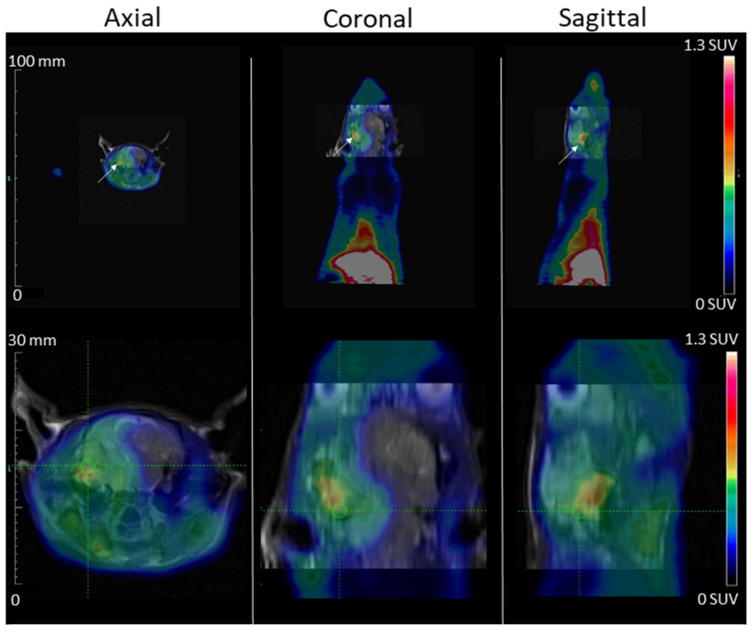
MDA-MB-231 tumor development in the rat brain cerebellum region after carotid injection is shown on MRI (day 52) and PET Scan (day 55)-fused images. PET images, acquired during the 2nd hour after [^68^Ga]Ga-NODAGA-K(Cy5)DKPPR injection, showed the fixation of [^68^Ga]Ga-NODAGA-K(Cy5)DKPPR at the anterior part of the animal (maximum field of view and brain view). The tumor is indicated with white arrows, the spatial scale is on the left, and the color scale (right) is expressed in SUV values.

Thanks to the intracardial injection metastasis model, MDA-MB-231 tumor development in the nude rat brain cerebellum region was visible on an MRI at d52. No tumor area was observed outside the cranium, and particularly, no tumor was observed in the lungs and mandibles, which are often the sites of secondary tumor development when cells are injected through the venous route. The PET scans exams were performed at d55 for [^68^Ga]Ga-NODAGA-K(Cy5)DKPPR (Figure 8). The high uptake of the radiotracer in the tongue was not surprising in view of its hyper vascularization. NRP-1 is a multifaceted vascular regulator: every cell of the vascular system expresses NRP-1, and NRP-1 functions in several signaling pathways critical for blood vessel development and function. By regulating TGFβ signaling in endothelial cells and PDGF signaling in VSMCs, NRP-1 has the potential to not only contribute to angiogenesis but, also, to regulate important vascular maturation events. The focus of studies on the expression of NRPs and their ligands in the epithelium has been carcinoma, but a few studies have described their roles in normal physiological tissues [26,27,28]. Although in a lower proportion compared to the tumor vascular network, the basal protein expression of NRP-1 in healthy tissues could have an impact on distribution. In humans, NRP-1 protein expression has indeed been detected in the uterus, liver, gastrointestinal tract, etc. [29]. The circulating NRP-1 levels in mice, rats, monkeys, and humans are 427 ± 77, 20 ± 3, 288 ± 86, and 322 ± 82 ng/mL [30].

The passage of the molecules targeting NRP-1 across the blood–brain barrier (BBB) will have to be verified by other approaches. In vivo*,* orthotopic tumor models such as glioblastoma will allow us to evaluate the passage of molecules through an intact BBB (small tumors). We will also be able to test the molecules in in vitro models of the BBB and brain–tumor barrier.

**Figure 9:**
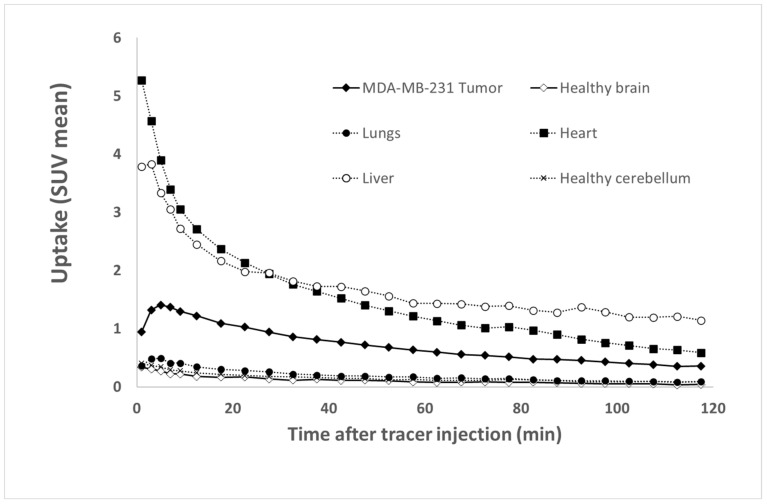
Tissue Time Activity Curves (TTACs) showing the fixation profiles of [^68^Ga]Ga-NODAGA-K(Cy5)DKPPR for tumors and various organs.

The Time Activity Curves (TTACs) obtained in vivo for the tumors and for different organs after the injection of [^68^Ga]Ga-NODAGA-K(Cy5)DKPPR at d55 are shown in Figure 9.

[^68^Ga]Ga-NODAGA-K(Cy5)DKPPR showed a rapid circulation through the heart and the liver from the first minutes after injection. The tumor uptake of [^68^Ga]Ga-NODAGA-K(Cy5)DKPPR decreased progressively with time and seemed to become stable later from the second hour. However, a very weak uptake of the healthy brain can be detected for this radiotracer, thus allowing discrimination of the tumor focus for this organ (see Supplementary Data Figure S10). Tumor and healthy brain normalized TTACs showed a specific radiotracer uptake in the tumor in the cerebral stage, with a plateau reached roughly 10 min after injection.

Indeed, 1 h after the injection of [^68^Ga]Ga-NODAGA-K(Cy5)DKPPR, the tumor uptake (SUV mean) was 7.6-fold higher than the surrounding healthy brain (SUV mean = 0.61 versus 0.08 for the tumor and healthy brain, respectively) (see Supplementary Data Figure S10).

## 3. Discussion

In this paper, we designed an original radiotracer for multimodalities of PET and fluorescence imaging applications and the targeting NRP-1 receptor. This receptor is overexpressed in angiogenic endothelial cells and in some tumors like glioblastoma and breast cancer, where it is associated with a poor prognosis. In our strategy, the targeting of NRP-1 allows us to target both vascularized brain metastases and avascular tumors. In our previous studies, we have shown that the KDKPPR peptide presented a good affinity for NRP-1 [22,24].

For PET modalities, the gallium-68 positron emitter was chosen as the radioisotope, allowing to obtain an efficient radiochemistry by a complexation reaction. To perform this complexation, NODAGA was used as the bifunctional chelator for its capacity with radiolabeled gallium-68 at room temperature. This is interesting for thermosensitive peptides. NODAGA is derivative of the hexadentate N_3_O_3_ NOTA chelator family. It is able to form more stable Ga complexes compared to NOTA thanks to the additional carboxylate function, allowing the best coordination with ^68^Ga^3+^ [31]. The NOTA chelator is the oldest and most successful chelator of gallium-68 and is considered to be the gold standard for Ga^3+^ complexation.

For fluorescent application, Cy5 was chosen due to its interesting photophysical properties, namely a fluorescent emission in the red region and a high molar extinction coefficient. In the future, we aim to perform fluorescence microscopy.

In our molecular design approach, we decided to link these 3 entities around a linker platform able to perform three links. The amino acid lysine was chosen for this role. The synthesis of the chelator–dye–peptide compound was performed with success in 6 steps on SPPS. To perform the in vitro experiment, the DKPPR peptide, scramble peptide (RPKPD), KDKPPR, peptide containing Cy5 dye K(Cy5)DKPPR and K(Cy5) RPKPD, nonradioactive reference ^nat^Ga-NODAGA-K(Cy5)DKPPR, and ^nat^Ga-NODAGA-K(Cy5) RPKPD were synthesized.

The compounds were synthesized by the Fmoc/tBu/Dde solid-phase strategy. The Dde group is an amino protective group that can be used orthogonally with both Fmoc and Boc protections [32]. The Dde group is cleaved with 3% hydrazine in DMF. Compared to Cy5-NHS, after conjugation of the peptides and NODAGA with Cy5, a decrease of ε could be observed by a factor of 4.7 for NODAGA-K(Cy5)DKPPR and 5.2 for NODAGA-K(Cy5)scramble, whereas an increase of φ_F_ was simultaneously observed (27% for Cy5-NHS, 61% for NODAGA-K(Cy5)DKPPR, and 79% for NODAGA-K(Cy5)scramble).

In previous studies, we have shown that molecules coupled to the ATWLPPR peptide had a good affinity for the NRP-1 protein [17,33]. ATWLPPR was specific for NRP-1, with an IC_50_ equal to 19 μM that increased after coupling (171 μM). In order to improve the selectivity, a new DKPPR peptide was designed and optimized by our team based on the screening methodology [23]. This peptide sequence was based on the sequence homology of the domain encoded by exon 8a of VEGF-165, and the amino acid lysine was added to allow its grafting without affecting the affinity. A docking analysis combined with biological evaluations demonstrated hydrogen bonds and π-π interactions between the NRP-1 protein and the DKPPR peptide. Using novel multimodal and multiscale approaches, we demonstrated the maintenance of the molecular affinity for the NRP-1 protein after the addition of a lysine residue at the *N*-extremity (KDKPPR peptide) but, also, after grafting KDKPPR to the nanoparticles [34]. No toxicity was observed in either an endothelial cell line (HUVEC) or the MDA-MB-231 cancer line, nor were any changes in the cell proliferation or metabolism observed for ^nat^Ga-NODAGA-K(Cy5)DKPPR. Our team has already demonstrated the great interest of peptide moieties targeting the NRP-1 protein overexpressed by endothelial cells with an angiogenic phenotype. This recognition resulted in a prolonged retention time into the endothelial cells lining the vasculature of xenotransplanted human glioblastomas, followed by incorporation into the tumor tissue [26,27,28,35]. In addition, in this study, we evaluated the affinity of the KDKPPR free peptide and conjugated the ^nat^Ga-NODAGA-K(Cy5)DKPPR peptide using SPR experiments (Table 2), SPR being a reference method.

All these in vitro results encouraged us to pursue them in vivo.

In the objective to use NODAGA-K(Cy5)DKPPR as a radiotracer, it was radiolabeled with gallium-68 in classical radiolabeling conditions (labeling at room temperature over 10 min). The optimization of radiolabeling allowed obtaining the radiotracer in a high radiochemical yield (RCY) and purity. Formation of the radioactive byproduct was limited by adding ascorbic acid due to its antioxidant rules. The radiolabeling conditions were similar to those used previously for [^68^Ga]Ga-NODAGA-RGD [36]. The process of radiosynthesis on the mAIO (Trasis^®^) synthesizer was efficient, robust, and reproducible (RCY = 90.3 ± 2.5% dc; *n* = 12) to produce [^68^Ga]Ga-NODAGA-K(Cy5)DKPPR.

Coupling a peptide targeting NRP-1 to a fluorophore and/or radiotracer is relatively little-developed, and there are few publications in the literature. Thoreau et al. coupled the peptide ATWLPPR as an NRP-1-targeting peptide with a platform containing four RGD to target αvβ_3_ integrin and Cy5-5 for fluorescent imaging application. It was evaluated in vivo in a mice model bearing a U87MG tumor. The dual targeting induced better binding and selectivity [37].

Perret et al. were the first to use peptide ATWLPPR for nuclear medicine application. To label ATWLPPR with ^99m^Tc for SPECT application, a benzoyl mercapto group was attached to the *N*-extremity of the peptide to do a tetradentate Tc complex [38]. A preclinical evaluation was realized on MDA-MB-231 tumor-bearing nude mice. Even if they could not obtain images due to low affinity and receptor concentrations and/or fast elimination of the peptide, this study described, for the first time, a radioligand targeting NRP-1. Wu et al. developed another radiotracer able to target both integrin αvβ_3_ with RDG and NRP-1 with ATWLPPR [21]. This heterodimeric compound was labeled with Fluor-18 by complexation into a NOTA chelator. It was evaluated by PET in vivo in a mice model bearing the U87MG tumor. The dual targeting of both integrin αvβ_3_ and NRP-1 resulted in a better selectivity and uptake and retention into the tumor. In the last few years, the development of multimodal imaging PET and fluorescence has emerged as a promising tool for the development of new diagnostics and treatment agents [39,40,41]. The access of fluorescent tools is very interesting for a better understanding and a better sensibility for in vivo evaluations.

## 4. Materials and Methods

### 4.1. Synthesis

All reactions involving cyanine5 compounds were performed in the dark. Preparative reverse-phase high-performance liquid chromatography (HPLC) was performed on SPOT PREP Liquid Chromatography (ARMEN Instrument, Saint-Avé, France) with a Pursuit 5-C18 column (5 μm, 21.2 × 150 mm; Varian). Pure compounds obtained were analyzed accordingly by LC-MS on a Shimadzu LCMS-2020 monitored by a Diode Array Detector SPD-M20A (Shimadzu, Marne la Vallée, France) using a Pursuit 5-C18 column (4.6 × 150 mm; Varian) (Varian, Agilent Technologies, Santa Clara, CA, USA). Absorption spectra were recorded on a Perkin-Elmer Lambda EZ210 (Perkin-Elmer, Courtaboeuf, France) double-beam UV–visible spectrophotometer. Fluorescence spectra were recorded on a Fluorolog-3 spectrofluorometer FL3-222 (Horiba Jobin Yvon, Longjumeau, France) with a thermostated cell compartment (25 °C) using a 450-W Xenon lamp. The fluorescence quantum yield (φ_F_) was determined using tetraphenyl porphyrin (TPP) solution in toluene as the fluorescence standard (φ_F_ = 0.11).

Unless otherwise stated, all chemicals were purchased as the highest purity commercially available and were used without further purifications. Cyanine5-NHS ester was purchased from Lumiprobe GmbH (Lumiprobe Gmb, Hannover, Germany). NODAGA-NHS ester was purchased from CheMatech (CheMatech, Dijon, France). The Fmoc-Arg(Pbf)-Wang resin, Fmoc-Asp(OtBu)-Wang resin, 9-fluorenyl-methoxy-carbonyl (Fmoc)-amino acids, and *N*,*N*,*N*′,*N*′-Tetramethyl-O-(1H-benzotriazol-1-yl)uronium hexafluorophosphate) (HBTU) were purchased from Iris Biotech GmbH (Iris Biotech GmbH, Marktredwitz, Germany). *N*-methylmorpholine (NMM), *N*-methylpyrrolidinone (NMP), trifluoroacetic acid (TFA), and triisopropylsilane (TIPS) were purchased from Alfa Aesar (Alfa Aesarn Haverhill, MA, USA). Hydrazine monohydrate, diisopropylethylamine (DIPEA), and Ga(NO_3_)_3_ were obtained from Sigma-Aldrich (Sigma-Aldrich, Taufkirchen, Germany). Hydrochloric acid (HCl; 36.5−38%) was purchased from Sigma-Aldrich (Sigma-Aldrich, St. Louis, MO, USA). Only Milli-Q water (0.18 MΩ cm) was used for the aqueous solution preparation.

#### 4.1.1. General Procedure of Peptide Synthesis

The side chains of aspartic acid and arginine were, respectively, protected by 5-tert-butyl ester (OtBu) and 2,2,4,6,7-pentamethyldihydrobenzofuran-5-sulfonyl (Pbf). Lysine was protected either by *N*-tert-butyloxy carbonyl (Boc) groups or (4,4-dimethyl-2,6-dioxocyclohex-1-ylidene)ethyl (Dde). H-K(Dde)D(OtBu)K(Boc)PPR(Pbf)-Wang resin was synthesized on a multichannel peptide synthesizer (Intavis AG, Köln, Germany), according to classical Fmoc/tBu solid-phase methodology, using the Fmoc-Arg(Pbf)-Wang resin on a 100-μmol scale. Double coupling was performed using a three-fold excess of *N*-Fmoc amino acid and activation reagents HBTU (3 equivalents), NMP (3 equivalents), and NMM (9 equivalents) in DMF. During the coupling of cyanine 5 and all the next steps, the light exposure was minimized to limit unwanted side reactions by sealing the vessel in aluminum foil. The final Fmoc protection or Dde protection was removed using piperidine or hydrazine monohydrate, respectively. The resin obtained was dried under vacuum and then cleaved using TFA/TIPS/water (92.5/5/2.5%) for 2 h. The acidic resin was filtered and washed with 2 mL of TFA and 50 mL of CH_2_Cl_2_. The filtrate was dried under vacuum and then purified according to the appropriate method described below. Peptides were purified by preparative reversed-phase (RP) HPLC and characterized by ESI^+^ mass spectrometry.

##### Synthesis of KDKPPR

The peptide was synthesized according to the general procedure described above. The crude product was further purified by precipitation in cold diethyl ether. Pure product was isolated as a white powder (32.5 mg, 44%). (ESI): *m*/*z* calcd for C_32_H_58_N_11_O_9_ [M + H]^+^, 740.43; found, 740 and [M + 2H]^2+^, 370.71; found, 371.

##### Synthesis of KPRKPD (i.e., Kscramble)

The peptide was synthesized according to the general procedure described above using a Fmoc-Asp(O*t*Bu)-Wang resin. The crude product was further purified by precipitation in cold diethyl ether. Pure product was isolated as a white powder (31 mg, 41.9%). (ESI): *m*/*z* calcd for C_32_H_58_N_11_O_9_ [M + H]^+^, 740.43; found, 740 and [M + 2H]^2+^, 370.71; found, 371.

##### Synthesis of Fmoc-K(Cy5)-OH

*N*-hydroxysuccinimide-activated cyanine5-NHS (75 mg, 0.12 mmol) and triethylamine (0.42 mmol, 50.3 μL) in 10-mL CH_2_Cl_2_ were added to Fmoc-Lys–OH and HCl (74 mg, 0.18 mmol) in a minimum of DMF in the dark under a nitrogen atmosphere. After stirring at room temperature for 24 h, the solvent was evaporated. The compound was purified by preparative HPLC with acetonitrile/(water, 0.1% TFA)(10/90) to 100% acetonitrile in 15 min, followed by isocratic acetonitrile for 10 min. Rt = 15.8 min. Pure product was isolated as a blue powder (66 mg, 65.5%). Identity and purity of the chelate were confirmed by ESI^+^ mass spectrometry and HPLC. (ESI): *m*/*z* calcd for C_53_H_62_N_4_O_5_ [M + H]^+^, 834.46; found, 834 and [M + 3H]^3+^, 278.82; found, 279.

##### Synthesis of NODAGA-K(Cy5)DKPPR

The peptide was synthesized according to the general procedure described above. After removing the Fmoc protection using piperidine, the coupling of NODAGA-NHS ester (1.2 equivalents) was realized using DIPEA (3 equivalents) in DMF for 12 h. After three cycles of Dde deprotection by hydrazine monohydrate (3% in DMF for 3 min), the coupling of cyanine5-NHS ester (1.2 equivalents) was realized using DIPEA (3 equivalents) in DMF for 12 h. After cleavage from the resin, the compound was precipitated in cold diethyl ether and purified by preparative HPLC in the same conditions as KDKPPR. Rt = 9.0 min. Pure product was isolated as a blue powder (42 mg, 27%). (ESI): *m*/*z* calcd for C_79_H_118_N_16_O_17_ [M + H]^+^, 1562.88; found, 1563 and [M + 2H]^2+^, 781.94; found, 782 and [M + 3H]^3+^, 521.62; found, 522 and [M + 4H]^4+^, 391.47; found, 391.

##### Synthesis of NODAGA-K(Cy5)RPKPD (i.e., NODAGA-K(Cy5)scramble)

The peptide was synthesized according to the general procedure described above using the Fmoc-Asp(O*t*Bu)-Wang resin. After removing the Fmoc protection using piperidine, the coupling of the NODAGA-NHS ester (1.2 equivalents) was realized using DIPEA (3 equivalents) in DMF for 12 h. After three cycles of Dde deprotection by a hydrazine monohydrate (3% in DMF for 3 min, the coupling of the cyanine5–NHS ester (1.2 equivalents) was realized using DIPEA (3 equivalents) in DMF for 12 h. After cleavage from the resin, the compound was precipitated in cold diethyl ether and purified by preparative HPLC in the same conditions as KDKPPR. Rt = 11 min. Pure product was isolated as a blue powder (44 mg, 28.2%). (ESI): *m*/*z* calcd for C_79_H_118_N_16_O_17_ [M + H]^+^, 1562.88; found, 1563 and [M + 2H]^2+^, 781.94; found, 782 and [M + 3H]^3+^, 521.62; found, 522 and [M + 4H]^4+^, 391.47; found, 391.

##### Complexation of NODAGA-K(Cy5)DKPPR with ^nat^Ga for In Vitro Assay

A small amount (0.5 μmol) of NODAGA peptide was dissolved in 700 μL of an ammonium acetate solution (0.1 M in H_2_O). Three hundred microliters of a ^nat^Ga(NO_3_)_3_ solution (0.04 M in H_2_O) were added and the pH adjusted to 4.5 with diluted HCl. The solution was then incubated for 30 min at 37 °C and directly purified by preparative HPLC using acetonitrile/water (0.1% TFA; 10/90) to a 100% acetonitrile gradient in 15 min, followed by isocratic acetonitrile for 10 min. Rt = 11.6 min. Pure product was isolated as a blue powder (83.17%). Identity and purity of the chelate were confirmed by ESI^+^ mass spectrometry and HPLC. (ESI): *m*/*z* calcd for C_79_H_115_GaN_16_O_17_ [M + H]^+^, 1628.78; found, 1629 and [M + 2H]^2+^, 814.89; found, 815 and [M + 3H]^3+^, 543.59; found, 544 and [M + 4H]^4+^, 407.94; found, 408.

##### Complexation of NODAGA-K(Cy5)RPKPD with ^nat^Ga for In Vitro Assay

The same procedure was followed to complex NODAGA-K(Cy5)RPKPD with Ga. Rt = 11.7 min. Pure product was isolated as a blue powder (82%). Identity and purity of the chelate were confirmed by ESI^+^ mass spectrometry and HPLC. (ESI): *m*/*z* calcd for C_79_H_115_GaN_16_O_17_ [M + H]^+^, 1628.78; found, 1629 and [M + 2H]^2+^, 814.89; found, 815 and [M + 3H]^3+^, 543.59; found, 544 and [M + 4H]^4+^, 407.94; found, 408.

##### Synthesis of K(Cy5)DKPPR

The peptide was synthesized according to the general procedure described above. Fmoc-Lys(Boc)-OH was replaced by Fmoc-Lys(Cy5)-OH. The crude product was further purified by preparative HPLC in the same conditions as KDKPPR. Rt = 14.5 min. Pure product was isolated as a blue powder (20 mg, 16.6%). Identity and purity of the chelate were confirmed by ESI^+^ mass spectrometry and HPLC. (ESI): *m*/*z* calcd for C_64_H_95_N_13_O_10_ [M + H]^+^, 1205.72; found, 1205 and [M + H]^2+^, 603.36; found, 603 and [M + 3H]^3+^, 402.57; found, 402.

##### Synthesis of K(Cy5)RPKPD (i.e., K(Cy5)scramble)

The peptide was synthesized according to the general procedure described above using the Fmoc-Asp(O*t*Bu)-Wang resin. Fmoc-Lys(Boc)-OH was replaced by Fmoc-Lys(Cy5)-OH. The crude product was further purified by preparative HPLC in the same conditions as KDKPPR. Rt = 14.5 min. Pure product was isolated as a blue powder (18 mg, 15%). Identity and purity of the chelate were confirmed by ESI^+^ mass spectrometry and HPLC. (ESI): *m*/*z* calcd for C_64_H_95_N_13_O_10_ [M + H]^+^, 1205.72; found, 1205 and [M + H]^2+^, 603.36; found, 603 and [M + 3H]^3+^, 402.57; found, 402.

### 4.2. Radiochemistry

HPLC analyses were run on a Waters system (2695eb pump, auto sampler injector, 2998 PDA detector, and NaI detector from Berthold (Bad Wildbad, Germany)) controlled by Empower Software (Orlando, FL, USA). Analyses were performed on a Pursuit XRs 5C18 (5 μm, 250 × 4.6 mm) from Agilent with an ACN/H_2_O/TFA mixture (proportions given in brackets) at 1 mL/min.

Approximatively 800 MBq of ^68^GaCl_3_ was eluted from a ^68^Ge/^68^Ga generator (Galliapharm; Eckert & Ziegler Europe) and eluted with 5 mL of a 0.1-N HCl solution. Radiosynthesis was carried out on a miniAllInOne (miniAIO) module from Trasis^®^.

#### 4.2.1. Radiosynthesis

The precursor NODAGA-K(Cy5)DKPPR kept in a stock solution of 1 mg/mL in deionized water. [^68^Ga]Ga-NODAGA-K(Cy5)DKPPR was prepared on the mAIO (Trasis, Ans, Belgium) synthesizer with a ^68^Ge/^68^Ga generator from Eckert and Ziegler eluted with 5 mL of the 0.1-M HCl solution and used without pre-purification of the eluate (Figure S11). To determine the radiolabeling efficiency, different parameters were screened, such as the amount of precursor, duration, and temperature (see Supplementary Data Figure S12). The best radiolabeling conditions found were the use of 50 µg of precursor NODAGA-K(Cy5)DKPPR with 10 µL of ascorbic acid 1 M diluted in a 0.8-M AcONa solution (1 mL) at 30 °C for 10 min. After incubation, this crude solution was purified by a HLB (Oasis) cartridge to isolate [^68^Ga]Ga-NODAGA-K(Cy5)DKPPR, formulated for injection in EtOH/NaCl 0.9% (10/90 *v/v*) and submitted for quality control. [^68^Ga]NODAGA-K(Cy5)DKPPR was produced with a decay-corrected (dc.) radiochemical yield (RCY) of 90.3 ± 2.5% (*n* = 12).

The radiochemical purity and specific activity of [^68^Ga]Ga-NODAGA-K(Cy5)DKPPR were evaluated with HPLC analyses using a reversed-phase C18 column (Pursuit XRs 5C18). The eluent consisted of 0.1% TFA in Milli-Q water (solvent A) and an acetonitrile (solvent B) gradient of 90–0% A and 10–100% B used over a period of 15 min with a flow rate of 1.0 mL/min.

#### 4.2.2. In Vitro Stability

The stability of [^68^Ga]Ga-NODAGA-K(Cy5)DKPPR was performed in various media, final formulated media, and plasma.

The final [^68^Ga]Ga-NODAGA-K(Cy5)DKPPR was kept at room temperature and analyzed by radio-HPLC at the end of the synthesis and 1 h, 2 h, and 3 h after manufacturing.

For the stability in plasma, 100 µL of [^68^Ga]Ga-NODAGA-K(Cy5)DKPPR was added to 400 µL of fresh rodent plasma and was incubated at 37 °C for 0, 30, 60, and 120 min. After incubation, the solutions were treated with 400 µL of acetonitrile to precipitate the proteins, vortexed for 1 min, and then centrifuged (2500 rpm, 980 G, 5 min). The supernatant layer was analyzed by analytical radio-HPLC.

#### 4.2.3. Determination of Distribution Coefficient (log D)

A sample of [^68^Ga]Ga-NODAGA-K(Cy5)DKPPR (50 µL, ~2 MBq) was added in a vial containing 1000 µL of 1-octanol and 950 µL of PBS. The vial was vortexed vigorously during 1 min and then centrifuged for 5 min for phase separation. The defined volume of each layer was measured with a γ-counter (Perkin Elmer, Wizard 2470). The distribution coefficient was expressed as the logarithm of the ration of the counts per minute measured in the 1-octanol phase to the counts per minute measured in the PBS phase in the same volume. The values reported are the average of 3 independent measurements (+/−SD), each performed in quintuplet.

### 4.3. Binding to Recombinant NRP-1 Protein

#### 4.3.1. Competitive Binding Assay

The binding of compounds for the NRP-1 protein was evaluated in terms of the half-maximal inhibitory concentration (IC_50_ values) through a competitive assay, as previously described [24]. VEGF-165, the natural ligand of NRP-1, was used as the positive control. Briefly, the surfaces of Maxisorp microplates (Thermo Fisher Scientific, France) were coated with NRP-1 recombinant chimeric proteins (Bio-techne, France). The binding of compounds to NRP-1 was assessed using biotinylated VEGF-165 (b-VEGF-165) (AcroBioSystem, Interchim, France) in the presence of heparin (Bio-techne, France) in competition or not with various concentrations of compounds or unlabeled VEGF-165 (Bio-techne, France) as the positive control. After 2 h of incubation at room temperature, the plates were washed, and the amount of bound b-VEGF-165 was stained with streptavidin horseradish peroxidase conjugate (Bio-techne, France) and assayed. The optical densities were measured at 450 nm using a Multiskan Ascent plate reader (Thermo Fisher Scientific, Finland). The results were expressed as the mean of the relative absorbance percentage to wells containing only blocking buffers of triplicate measurements. The affinities were estimated as the IC_50_ values (i.e., the concentration of compounds that displaced 50% of β-VEGF-165 binding) calculated using the one-site fit log-IC_50_ nonlinear analysis regression of GraphPad Prism 6 software (v 6.05, USA).

#### 4.3.2. Surface Plasmon Resonance (SPR)

The equilibrium dissociation constant (K_D_) was evaluated using surface plasmon resonance, based on Gries et al. [34]. All experiments were performed at 25 °C using the BIACORE T200 instrument (GE Healthcare Biacore, Uppsala, Sweden). Sensor surfaces and other Biacore consumables were purchased from GE Healthcare Biacore (USA). IgG1 and recombinant human/rat NRP-1 proteins were purchased from Bio-techne (France).

The CM5 Sensor Chip was activated by using sulfo-NHS/EDC (1-ethyl-3-[3-dimethylaminopropyl] carbodiimide) solution (0.2 µM) in PBS (phosphate-buffered saline) with 0.05% (*v/v*) P20 surfactant, pH 7.4. Ligands were subsequently immobilized on the chip: recombinant rat NRP-1 protein (MW: 119 kDa) in formate buffer (pH 3.0) or IgG1 (as the reference surface, MW: 26.6 kDa) in sodium acetate buffer (pH 4.0) at a concentration of 50 μg/mL and a flow rate of 5 μL/min during 10 min. The immobilization levels were 4000 resonance units (RU) for IgG1 and 10,000 RU for rat NRP-1 proteins. The chip was then blocked with 1 M of ethanolamine, pH 8.5.

^nat^Ga-NODAGA-Cy5, KDKPPR, and KRPKPD (4 mM) were dissolved in ultrapure water and diluted in running buffer (0.60–10 µM). They flowed at a rate of 30 µL/min for 300 s at 25 °C. The regeneration of surfaces was achieved using two injections of HCl 5 mM at 100 µL/min during 5 s, followed by a 300-s buffer flow. Sensorgrams were corrected for signals from the reference surface (blank subtracted sensorgram). The equilibrium response was recorded 5 s before the end of the compound injection (time window: 5 s). VEGF-165, a natural ligand of NRP-1, was used as the positive control. The K_D_ value was determined by fitting the response (RU) versus analyte concentration (µM) curve to a simple 1:1 interaction model with the BiaEvaluation 3.2 software (GE Healthcare). KD values represented the mean of two independent experiments.

### 4.4. Cell Culture

MDA-MB-231 (ATCC^®^ HTB-26™) (hormone-independent and triple-negative breast cancer cell line) was grown in RPMI 1640 medium (Merck, France) supplemented with 2.5-mM L-Glu, 100-U·mL^−1^ penicillin, 100-μg·mL^−1^ streptomycin, and 10% FCS.

HUVEC (LONZA, Belgium) (Human Umbilical Vein Endothelial Cells) was grown and used until passage 5 in endothelial basal medium supplemented with the EGM™-2 SingleQuots^®^ supplement containing FBS, hFGF-B, VEGF, R3-IGF, rhEGF, ascorbic acid, hydrocortisone, heparin, and GA (Gentamicin/Amphotericin-B) (Lonza, Belgium).

Cell cultures were performed under standard conditions (5% CO_2_ at 37 °C under 80% humidified air), and cells were negative for the mycoplasma detection assay (MycoSensor QPCR assay, Agilent, France).

#### 4.4.1. NRP-1 Expression of Cells

MDA-MB-231 and HUVEC were grown until subconfluence. Briefly cells were washed 3 times with ice-cold PBS before lysis using the RIPA buffer supplemented by complete (Roche, Germany) and antiprotease cocktails 2 and 3 (Sigma-Aldrich, St. Quentin Fallavier, France). After 30 min of incubation at 4 °C, cell lysates were harvested and passed through a 23 G needle. Protein extracts were kept at −80 °C before the evaluation of the protein concentration using the bicinchoninic acid method (Pierce, France). Fifty micrograms of protein were loaded onto 10% polyacrylamide gel electrophoresis, and migration was performed at 50 V over 30 min, followed by 2 h at 110 V. Protein transfer onto a PVDF membrane was performed using the “Mixed MW turbo” program of the transblot system (Bio-Rad, France) (7 min/2.5 A/25 V). The membrane was cut into 2 pieces to separate low MW protein and high MW protein after rouge ponceau labeling. Membranes were blocked with TBST + 5% nonfat milk during 1 h and incubated overnight at 4 °C with 1/1000 diluted anti-NRP-1 rabbit (D39A5, Ozyme, France) or anti-actin rabbit (4970S, Ozyme, France) antibodies. Secondary HRP-linked antibody (7074S, Ozyme, France) was diluted to 1/1000, and the revelation was performed using the ECL Western blotting detection system (Sigma-Aldrich, France) following the instructions of the manufacturer. Images were recorded on LAS-4000 (Fujifilm Life Science, USA), and the optical density of each band was evaluated using Multigauge 3.0 software (Fujifilm Life Science, USA).

#### 4.4.2. Metabolic Activity

Cell survival of MDA-MB-231 and HUVEC incubated with the NRP-1-targeting or nontargeting peptide or ^nat^Ga-NODAGA-conjugated compounds were investigated using a MTS assay. Briefly, cells were plated into 96-well plates at a density of 10,000 or 3500 cells.cm^−2^, respectively, and incubated at 37 °C for 2/3 days. Old culture media were discarded, and cells were exposed to 0–10 µM of the compounds. After 4 h or 24 h, cells were washed three times with HBSS and were further incubated in culture medium for 24 h. The metabolic activity of the cells was evaluated using [3-(4,5-dimethylthiazol-2-yl)-5-(3-carboxymethoxyphenyl)-2-(4-sulfophenyl)-2H-tetrazolium] salt according to the supplier’s recommendations (CellTiter 96^®^ AQueous One Solution Reagent, Promega). The absorbance of formazan crystals solubilized in culture media was evaluated at 492 nm using a Multiskan Ascent plate reader (Thermo Fisher Scientific, Finland). The result was given as the mean of 3 independent experiments performed in triplicate and expressed as a percentage of variation from control cells.

#### 4.4.3. Proliferation Assay

The proliferation of MDA-MB-231 and HUVEC cells were investigated using a label-free, noninvasive IncuCyte^®^ S3 live-cell imaging system (Sartorius, Germany). Cells were plated as described for metabolic activity placed in an incubator (Binder) at 37 °C. Cells were photographed at 2-h intervals using a 20× objective over several days. After 3 days, cells were exposed to 0–10 µM of the compounds until the end of the experiment. Phase–object confluence was calculated at each time point based on the segmentation of high-definition phase-contrast images using IncuCyte S3 2018B software and expressed in percentages. Experiments were performed in triplicate.

Cell doubling time (*DT*) was calculated using the following Equation (1):

(1) $$DT=\frac{\left(Tend-Tstart\right)Ln2}{LnCend-LnCstart}$$

where *Tstart*/*Tend* are the times at the start/end of the exponential growth phase. *Cstart*/*Cend* are the percentage of confluence of the cells at the start/end of the exponential growth phase.

### 4.5. Animal Care

All in vivo experiments were performed in accordance with the European Community animal care guidelines (Directive 2010/63/EU) for the use of experimental animals with respect to the 3 Rs’ requirements for animal welfare and carried out by competent and authorized persons in a registered establishment (establishment number D-54-547-03 issued by the Departments of Veterinary Services). All animal protocols were submitted to an ethics committee (Comité d’Ethique Lorrain en Matière d’Expérimentation Animale, CELMEA, French Ethical Committee number 66) for evaluation in accordance with the national regulations (research project APAFIS #19041 was approved by the French Ministry of Research).

Animals were kept under standard conditions (T = 24 °C ± 1 °C, hygrometry 50% ± 10%, a controlled 12-h light–dark cycle with ventilated cages, including filter tops) and had free access to standard food and water.

Eight-week-old nude female rats (Hsd: RH-Foxn1rnu) weighing 100–110 g were purchased from Charles River Laboratories (Charles River Laboratories, Saint Germain Nuelles, France).

#### 4.5.1. Brain Metastasis Model

Breast cells from the MDA-MB-231 lineage (MDA-MB-231 (ATCC^®^ HTB-26)) were induced by the vascular injection of 2.5 × 10^5^ cells resuspended in a volume of 50 µL of RPMI medium (Merck, Molsheim, France) into the animal’s internal carotid.

#### 4.5.2. MRI Experiment

Weekly longitudinal follow-ups of the tumor growth were conducted by MRI to detect metastatic outbreaks in the animal’s brain. The evolution of the metastatic focus was monitored by MRI imaging on a weekly basis for 8 weeks. MRI was initiated 10 days after tumor cell injection and followed a week later to detect brain lesions. A classic T2-weighted (T2w) morphological sequence was performed to validate the tumor development. This examination was done under general gassing anesthesia.

The MRI was performed on a 3T scanner (Prisma, Siemens Healthineers^®^, Erlangen, Germany) using an 8-channel volume coil dedicated to rats (Rapid Biomedical GmbH^®^, Rimpar, Germany). The MRI was initiated 10 days after tumor cell injection and followed a week later to detect brain lesions. Animals were anesthetized with isoflurane, and respiration was monitored and maintained constant throughout the experiment. Anatomical T2w images, covering from the frontal lobe to the posterior fossa, were acquired using a Turbo Spin-Echo (TSE) sequence (repetition time (TR)/echo-time (TE) = 2500/61 ms, Echo Train Length (ETL) = 6, flip angle = 90°, 20 slices, field of view (FOV) = 49 × 49 mm^2^, matrix size = 192 × 192, voxel size = 255 × 255 × 1000 μm^3^, Number of Scan Averages (NSA) = 8, and scan time = 10 min 37 s).

#### 4.5.3. PET Acquisition and Analysis

PET recordings were obtained with a camera dedicated to small animal studies (Inveon, Siemens Preclinical Solutions, Knoxville, TN, USA). After rats anesthetized with isoflurane, 50 MBq of [^68^Ga]Ga-NODAGA-K(Cy5)DKPPR (1st day) were injected as a bolus via a lateral tail vein.

For [^68^Ga]Ga-NODAGA-K(Cy5)DKPPR: 120-min list-mode acquisitions were initiated a few seconds prior to tracer injection, and the acquired PET data were subsequently reconstructed in 27 consecutive frames (5 frames of 120-s duration followed by 22 frames of 5-min duration) using the 3D ordered-subsets expectation maximization algorithm (OSEM3D, 4 iterations, 16 subsets, and 1 zoom) together with scatter and attenuation corrections based on transmission source measurements. No post-filter or regularization was applied after the reconstruction. The final voxel size was 0.8 × 0.8 × 0.8 mm^3^.

The uptake values are expressed in SUV (Standardized Uptake Values), which are standardized fixation values relative to the animal’s weight and injected activity. Tissue time activity curves (TTACs) for tumors and various organ activities were determined by average SUVs in volumes of interest (VOI), which were drawn with dedicated software (Inveon Research Workplace 4.1, Siemens^®^, Knoxville, USA) on static images encompassing the whole acquisition [35,36]. Ellipsoid VOIs were drawn within the tumor, contralateral healthy brain (a same symmetrical VOI in the tumor and contralateral healthy brain), cerebellum (area with a low uptake of tracers used to make a normalization ratio), lungs, heart, and liver. The tumor VOI was automatically segmented with 50% of the maximum voxel value threshold [35,36]. At last, the tumor and healthy brain were normalized to the cerebellum to assess the contrast evolution of the segmented areas.

#### 4.5.4. Immunohistological Analysis

Just after PET imaging, the rat was euthanized, and the tumor in the brain tissue was fixed in formalin and embedded into paraffin. On 5-µm tumor sections, anti-NRP-1 (rabbit monoclonal, 1/800, ab81321) Abcam staining was performed to detect the tumoral cell region and to confirm NRP-1 receptor expression. Each primary antibody was incubated with the tumor sections one night at 4 °C. The tumor sections were incubated with a secondary antibody (N-Histofine Universal Immuno-peroxidase Polymer Anti-Rabbit Ref414341F, Nichirei Biosciences) that was revealed by a peroxidase Kit (ImmPACT Novared Peroxidase substrate kit HRP SK-4805). Finally, the sections were counterstained with Mayer’s hematoxylin. Images of the tumors were captured using a Nikon Model Eclipse E600 microscope.

### 4.6. Statistical Analysis

All in vitro experiments were realized in triplicate and expressed as the percentage of variation from control cells. The results were given as the mean ± standard deviation (SD). Differences among the groups were evaluated using a variance analysis (ANOVA) followed by Bonferroni’s test (GraphPad Prism 6.0 software, San Diego, CA, USA). A value of *p* < 0.05 was considered statistically significant. * Versus control cells.

## 5. Conclusions

The synthesis of a new gallium-68 radiolabeled peptide to target NRP-1 was successfully achieved. The KDKPPR peptide was coupled with gallium-68 anchored into a bifunctional NODAGA chelating agent, as well as Cy5 for fluorescence detection. After the coupling of the peptide and NODAGA, the Cy5-NHS absorbance spectra did not change. However, ε decreased drastically. An enhancement of φ_F_ could be observed up to 61% for ^nat^Ga-NODAGA-K(Cy5)DKPPR and 79% for ^nat^Ga-NODAGA-K(Cy5)RPKPD, highlighting the fact that the NODAGA peptide improved the water solubility of Cy5. [^68^Ga]Ga-NODAGA-K(Cy5)DKPPR had a radiosynthesized efficiency and showed a hydrophilic property (log D = −1.86) and high in vitro stability (>120 min).

Using novel multimodal and multiscale approaches, we first validated the molecular affinity of this new chelated radiotracer to KDKPPR for the NRP-1 protein. In vitro, we observed no toxicity. The preliminary in vivo results also showed promising results, with a high contrast between the healthy brain and metastatic foci for [^68^Ga]Ga-NODAGA-K(Cy5)DKPPR.

## Figures and Tables

**Table 1:** Molar extinction coefficient (ε) and fluorescence quantum yield (φ_F_) of ^nat^Ga-NODAGA-K(Cy5)DKPPR, ^nat^Ga-NODAGA-K(Cy5)RPKPD, K(Cy5)DKPPR, K(Cy5)RPKPD, and Cy5-NHS in water (λ_ex_ = 590 nm).

Compound	ε (L mol^−1^ cm^−1^)	φ_F_ (%)
^nat^Ga-NODAGA-K(Cy5)DKPPR	44,552	61
^nat^Ga-NODAGA-K(Cy5)RPKPD	39,870	79
K(Cy5)DKPPR	53,757	35
K(Cy5)RPKPD	49,062	26
Cy5-NHS	209,000	27

**Table 2:** Binding to the recombinant NRP-1 protein by SPR. Dissociation constant K_D_ of the complex formed between NRP-1 and the compounds was calculated using the titration curves of the kinetic curve (response (RU) by function of the concentration (µM)) using the BiaEvaluation 3.2 software (GE Healthcare, Uppsala, Sweden).

Compounds	K_D_ +/− SD (µM)
KDKPPR	7 ± 2
K(Cy5)DKPPR	5 ± 1
^nat^Ga-NODAGA-K(Cy5)DKPPR	4 ± 2

**Table 3:** Doubling-time values (Time (h) ± SD) of the MDA-MB-231 and HUVEC cells exposed to NRP-1-targeting or nontargeting compounds (0–10 µM) calculated during the exponential phase of cell growth.

Compounds	Concentration (µM)	Doubling-Time Values (Time (h) ± SD)	
		MDA MB 231	HUVEC
Control	-	34 ± 2	29 ± 3
KDKPPR	1	32 ± 1	29 ± 2
	10	33 ± 2	30 ± 2
K(Cy5)DKPPR	1	34 ± 3	30 ± 6
	10	32 ± 1	28 ± 3
^nat^Ga-NODAGA-K(Cy5)DKPPR	1	31 ± 1	30 ± 3
	10	32 ± 1	28 ± 2
K(Cy5)RPKPD	1	32 ± 1	30 ± 1
	10	32 ± 2	30 ± 3
^nat^Ga-NODAGA-K(Cy5)RPKPD	1	33 ± 1	27 ± 1
	10	33 ± 3	28 ± 1

## Supplementary Materials

The following are available online: Figure S1. Analytical radio-HPLC profile of the [^68^Ga]Ga-NODAGA-K(Cy5)DKPPR radiolabeling precursor. Radiochemical purity of [^68^Ga]Ga-NODAGA-K(Cy5)DKPPR was ≥ 95% (*R*t = 8.9 min (identical retention time compared to the standard fig SX +/− 10%), Pursuit XRs 5C18, 5µm, 250 × 4.6mm, 10–100% ACN in gradient conditions in 15 min, 1.0 mL·min^−1^. Figure S2. Analytical UV-HPLC profile of the ^nat^Ga-NODAGA-K(Cy5)DKPPR nonradioactive reference at 640 nm (*R*t = 9.0 min, Pursuit XRs 5C18, 5 µm, 250 × 4.6 mm, 10–100% ACN in gradient conditions in 15 min, 1.0 mL·min^−1^). Figure S3. Stability studies of [^68^Ga]Ga-NODAGA-K(Cy5)DKPPR determined by radio-HPLC analyses. Figure S4. Sensorgrams (left) and titration (right) curves corresponding to the interactions between the rat NRP-1 and (A) KDKPPR peptides and (B) K(Cy5)DKPPR. Briefly, IgG1 and recombinant rat NRP-1 were immobilized on two surfaces of a CM5 sensor chip. Peptides (0.60–10 µM) were injected at a flow rate of 30 µL·min^−1^ at a temperature of 25 °C. Data were recorded and presented as the response (RU) as a function of time (s) after double referencing: subtraction of signal obtained on the reference IgG1 surface and subtraction of the buffer. These sensorgrams (left side) were used to draw titration curves (right side) by plotting responses recorded 5 s before the end of injection as a function of the analyte concentrations. Figure S5. Sensorgrams showing the responses of scrambled peptides and VEGF (natural ligand) on the recombinant NRP-1 protein by surface plasmon resonance (SPR). NRP-1 was immobilized on the CM5 surface. The AIgG1-Fc-immobilized surface was used as the reference surface. Peptides were injected on the NRP1 and IgG1-Fc surfaces at 10 and 30 µM, and VEGF was injected at concentrations ranging from 12.5 nM to 400 nM. Data were recorded and presented as the response (RU) in the function of time (s) after double subtraction of the signal obtained for buffer injection and on the IgG1-Fc reference surface. (A–D) represent the binding of KRPKPD, K(Cy5)RPKPD, ^nat^Ga-NODAGA-K(Cy5)RPKPD, and VEGF, respectively. Figure S6. Relative expression of NRP-1 by Western blotting. Upper: Protein extracts were submitted to Western blotting. Figure S7. Histological brain slices made in paraffin following formalin fixation and NRP-1 marking. Scale: 100 µm and 50 µm. anti-NRP-1 (rabbit monoclonal, 1/800, ab81321, Abcam) staining was performed to evaluate the NRP-1 tumor expression. See Section 4.5.4 for the complete immunological protocols. Figure S8. Effect of NRP-1-targeting compounds onto cellular metabolic activity. MDA-MB-231 (upper) and HUVEC (lower) were exposed to 0–10 µM of compounds for 4 h (left) or 24 h (right). The viability of the cell was assessed using the MTS assay. The results were presented as the mean ± SD (*n* = 3). Figure S9. Morphology of MDA MB 231 (left) and HUVEC (right) exposed to 10-µM NRP-1-targeting or nontargeting compounds during 3 or 2 days, respectively. Objective 20×. Scale bar = 50 µm. Figure S10. Tissue Time Activity Curves (TTAC) showing the interesting fixation profile of [^68^Ga]Ga-NODAGA-K(Cy5)KDKPPR for the tumor after normalization with a healthy cerebellum or healthy brain. Figure S11. Graphical representation of a single-use kit for the automated radiosynthesis of [^68^Ga]Ga-NODAGA-K(Cy5)DKPPR on mAIO (Trasis). Figure S12. Result of radiochemical optimization for [^68^Ga]Ga-NODAGA-K(Cy5)DKPPR synthesis. (A) Influence of the amount of precursor was evaluated by adding various quantities of precursor NODAGA-K(Cy5)DKPPR (8,15, 30, and 50 µL) from a stock solution of 1 mg·mL^−1^ at 30 °C for 10 min (*n* ≥ 3). (B) Influence of the temperature was evaluated with 15 µg of precursor NODAGA-K(Cy5)DKPPR diluted in 0.8-M AcONa 1 mL for 10 min (*n* ≥ 3). (C) Influence of the reaction time was evaluated with 15 µg of precursor NODAGA-K(Cy5)DKPPR diluted in 0.8-M AcONa 1 mL at 30 °C (*n* ≥ 3).

## Sample Availability

Samples of the compounds are not available.
