# Supplementary material for: Preliminary Study of New Gallium-68 Radiolabeled Peptide Targeting NRP-1 to Detect Brain Metastases by Positron Emission Tomography
Source: Molecules. 2021 Nov 30;26(23):7273. doi: 10.3390/molecules26237273 (PMC8659110; doi:10.3390/molecules26237273)
Supplement: Supplementary file 1 [file molecules-26-07273-s001.zip › molecules-1434125-supplementary.pdf]

Supporting Information

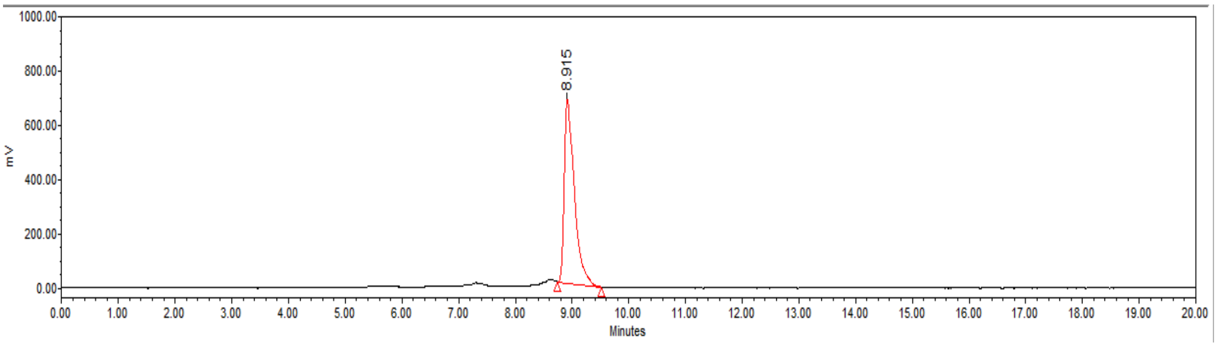

Figure S1. Analytical radio-HPLC profile of [^{68}Ga]Ga-NODAGA-K(Cy5)DKPPR radiolabeling precursor. Radiochemical purity of [^{68}Ga]Ga-NODAGA-K(Cy5)DKPPR was ≥ 95% (*R*t = 8.9 min (identical retention time compared to standard fig SX +/- 10%), Pursuit XRs 5C18, 5µm, 250x4.6mm, 10-100% ACN in gradient condition in 15 min, 1.0 mL.min^{-1}).

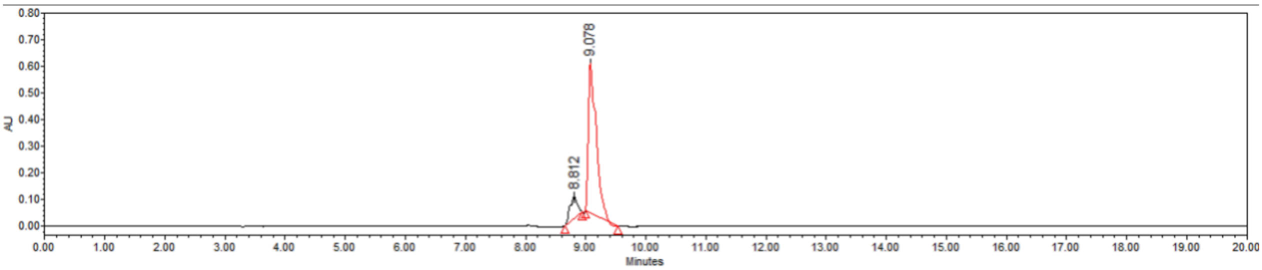

Figure S2. Analytical UV-HPLC profile of ^{nat}Ga-NODAGA-K(Cy5)DKPPR non-radioactive reference at 640 nm (*R*t = 9.0 min, Pursuit XRs 5C18, 5µm, 250x4.6mm, 10-100% ACN in gradient condition in 15 min, 1.0 mL.min^{-1}).

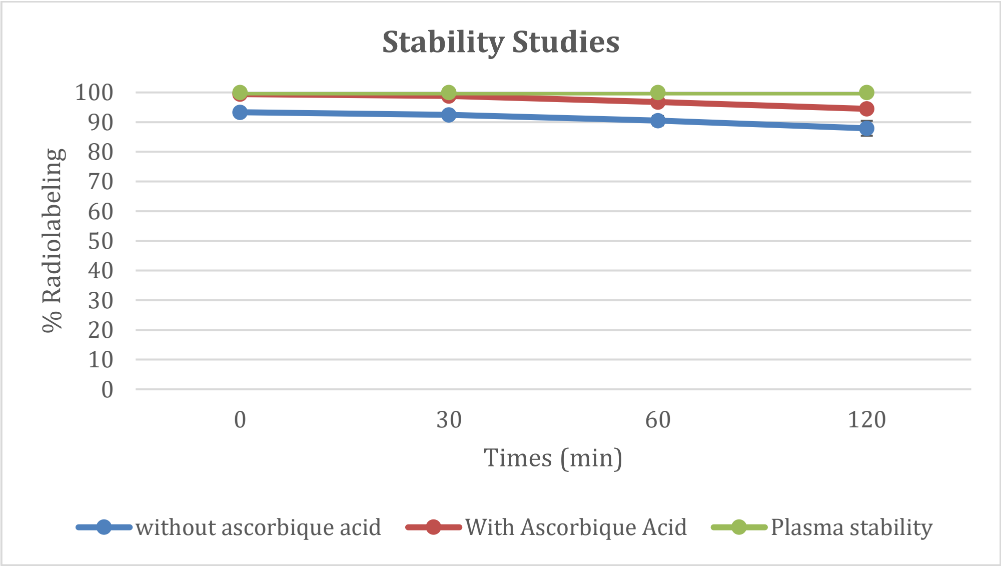

Figure S3. Stability studies of [^{68}Ga]Ga-NODAGA-K(Cy5)DKPPR determined by radio-HPLC analyses.

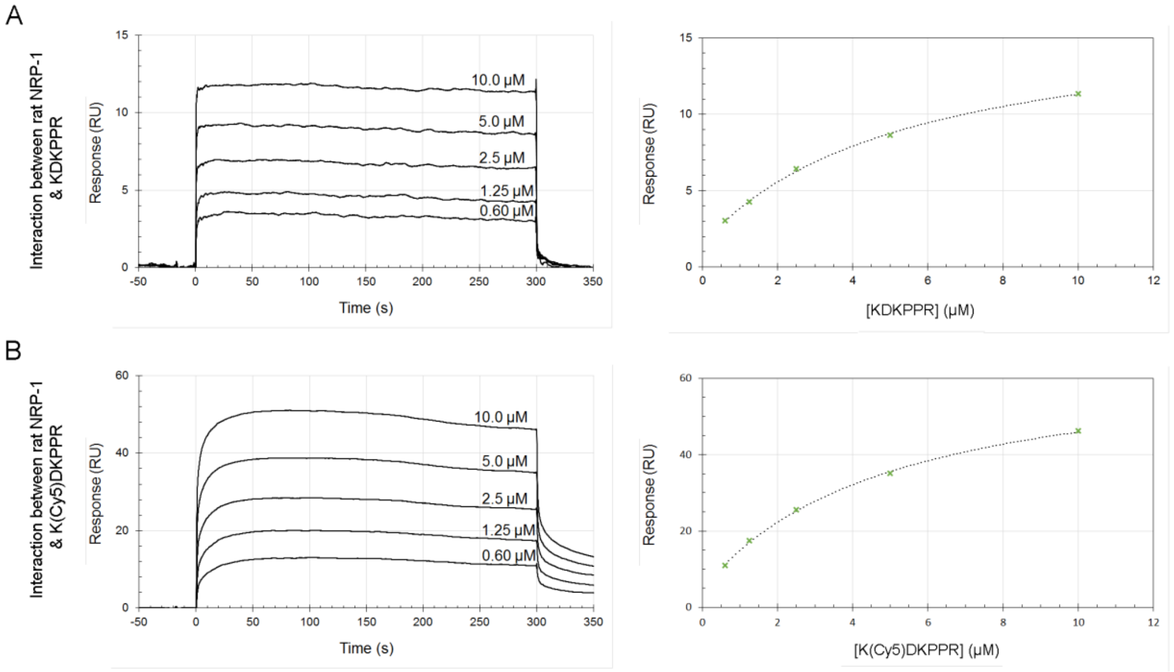

Figure S4. Sensorgrams (left) and titration (right) curves corresponding to the interaction between rat-NRP-1 and (**A**) KDKPPR peptide, (**B**) K(Cy5)DKPPR. Briefly, IgG1 and recombinant rat NRP-1 were immobilized on two surfaces of a CM5 sensor chip. Peptides (0.60 to 10 µM) were injected at a flow rate of 30 µL.min^{-1} at a temperature of 25°C. Data were recorded and presented as response (RU) as a function of time (s) after double referencing: subtraction of signal obtained on the reference IgG1 surface and subtraction of buffer. These sensorgrams (left side) were used to draw titration curves (right side) by plotting responses recorded 5 s before the end of injection as a function of analyte concentrations.

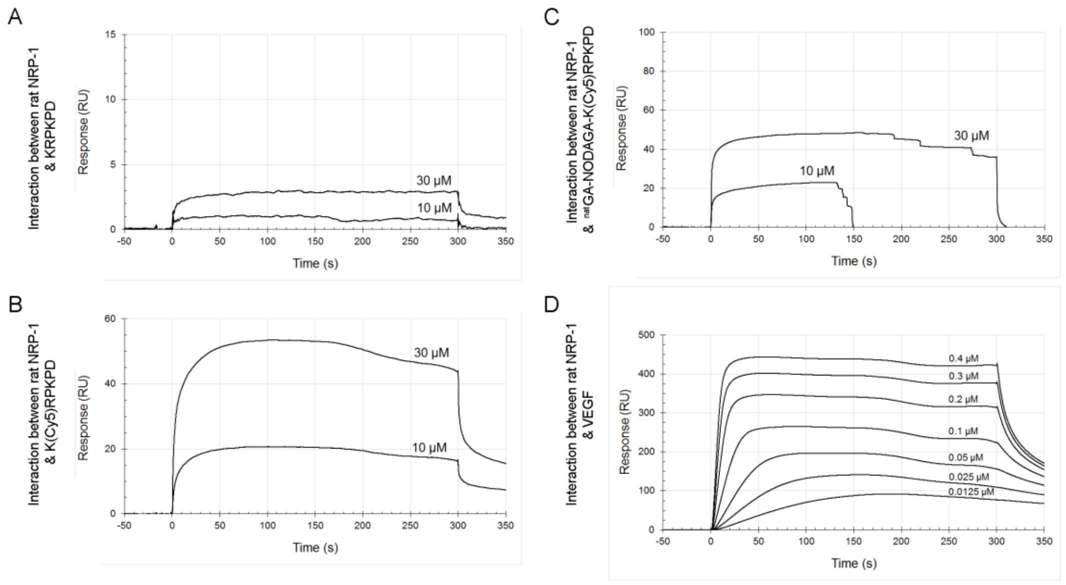

Figure S5. Sensorgrams showing the responses of scrambled peptides and VEGF (natural ligand) on recombinant NRP-1 protein by surface plasmon resonance (SPR). NRP-1 was immobilized on a CM5 surface. IgG1-Fc immobilized surface was used as the reference surface. Peptides were injected on the NRP1 and IgG1-Fc surfaces at 10 and 30 µM, and VEGF was injected at concentrations ranging from 12.5 nM to 400 nM. Data were recorded and presented as response (RU) in function of time (s) after double-subtraction of signal obtained for buffer injection and on the IgG1-Fc reference surface. A, B, C and D represent the binding of KRPKPD, K(Cy5)RPKPD, ^{nat}Ga-NODAGA-K(Cy5)RPKPD and VEGF respectively.

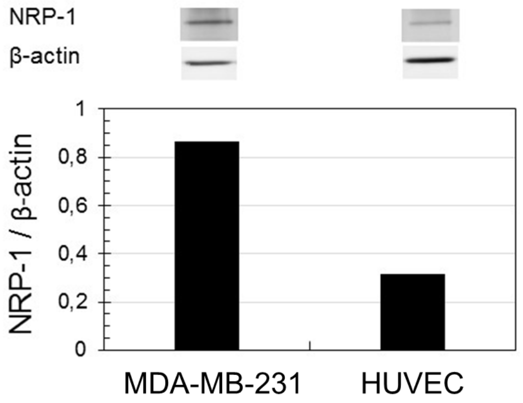

Figure S6. Relative expression of NRP-1 by western blotting. Upper: Protein extract were submitted to western blotting.

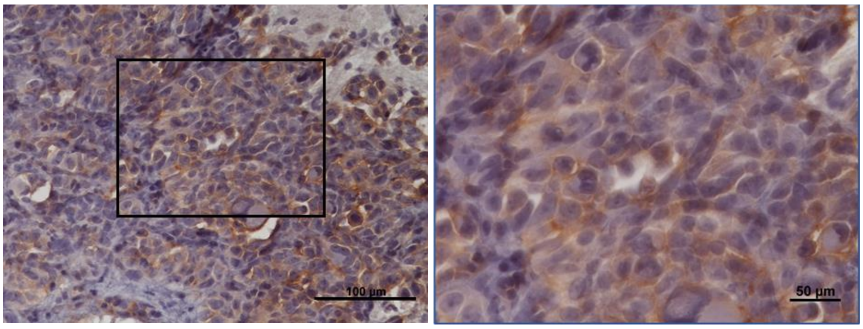

Figure S7. Histological brain slices made in paraffin following formalin fixation and NRP-1 marking. Scale: 100 µm, 50 µm. anti-NRP-1 (rabbit monoclonal, 1/800, ab81321, Abcam) staining was performed to evaluate NRP-1 tumor expression. See 4.5.4 for complete immunological protocol.

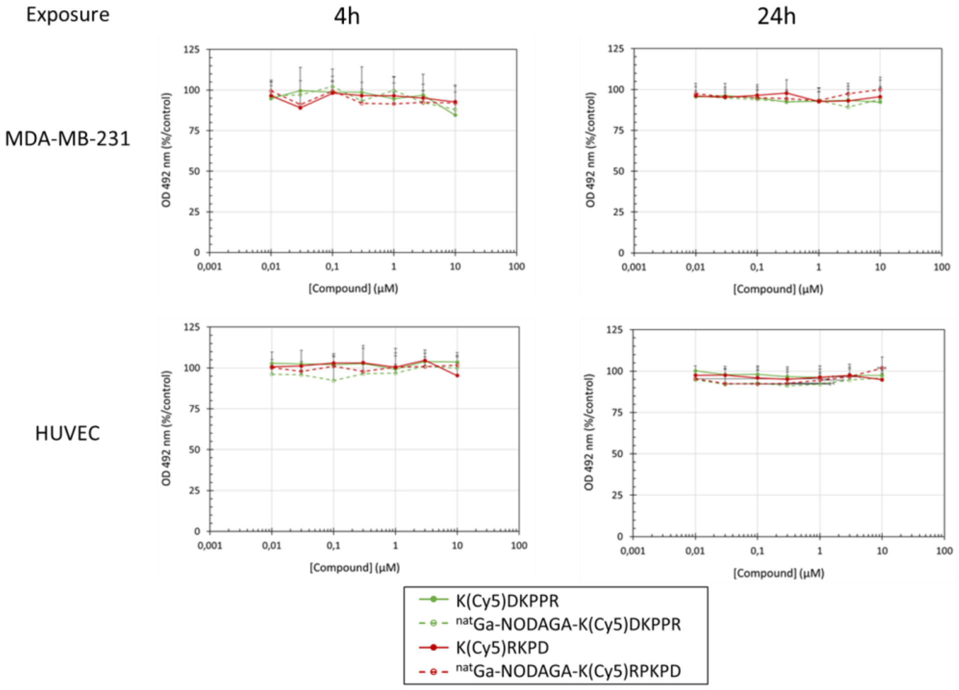

Figure S8. Effect of NRP-1 targeting compounds onto cellular metabolic activity. MDA-MB-231 (upper) and HUVEC (lower) were exposed to 0-10 µM of compounds during 4 h (left) or 24 h (right). The viability of cell was assessed using MTS assay. Results were presented as mean ± SD (n=3).

Compounds (Name)	MDA MB 231	HUVEC
Control	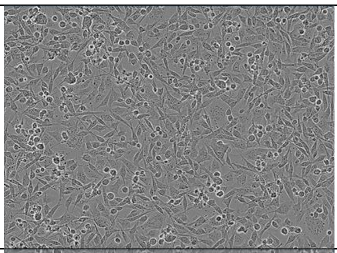	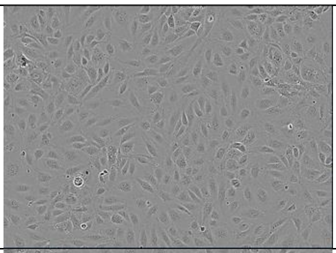
KDKPPR	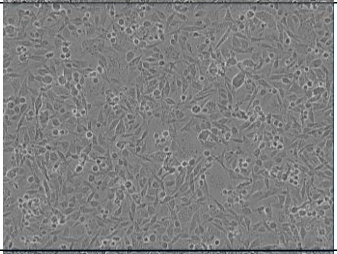	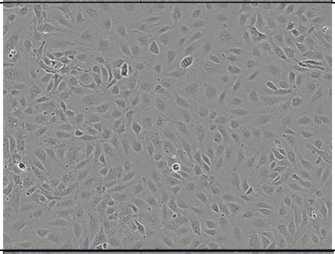
K(Cy5)DKPPR	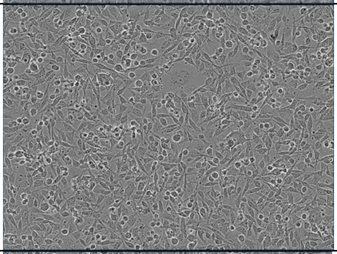	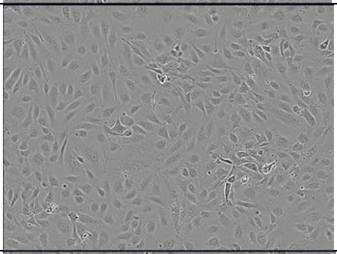
^{nat}Ga-NODAGA-K(Cy5)DKPPR	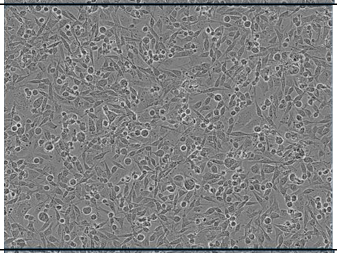	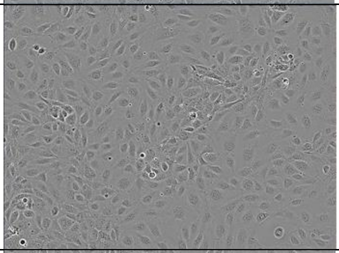
K(Cy5)RPKPD	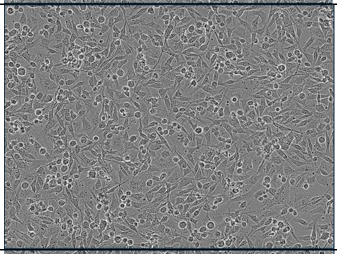	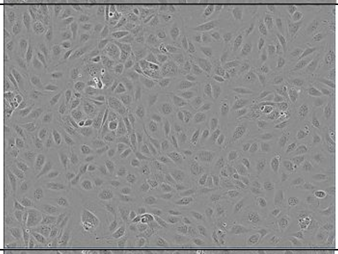
^{nat}Ga-NODAGA-K(Cy5)RPKPD	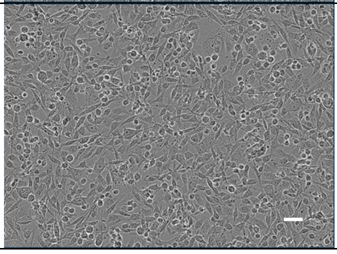	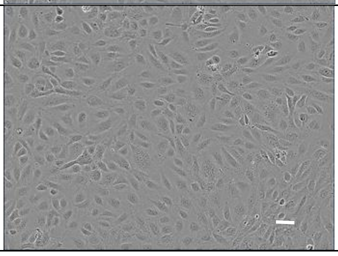

Figure S9. Morphology of MDA MB 231 (left) and HUVEC (right) exposed to 10 μM NRP-1 targeting or non-targeting compounds during 3 or 2 days respectively. Objective X20. Scale bar = 50 μm.

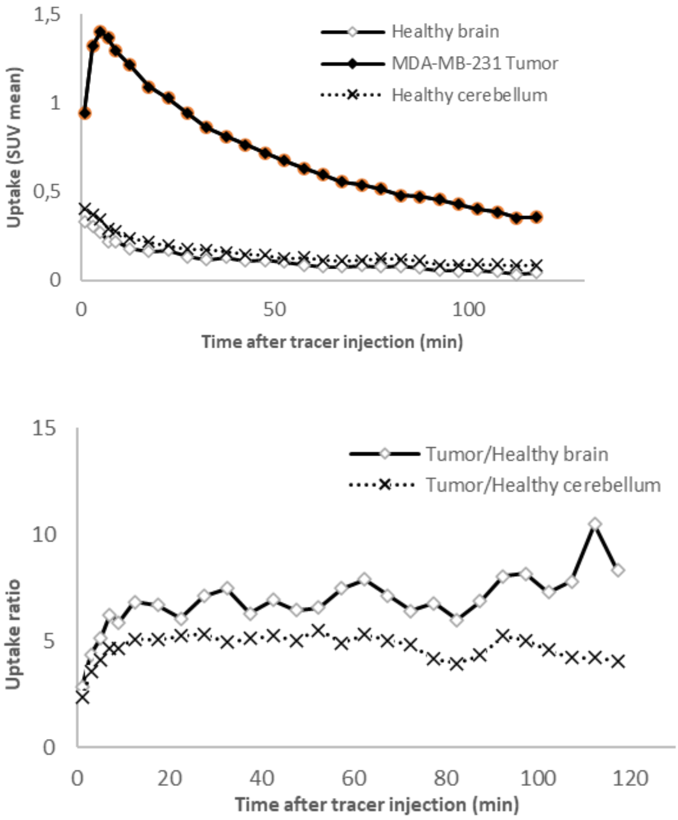

Figure S10. Tissue Time Activity Curves (TTAC) showing the interesting fixation profile of [^{68}Ga]Ga-NODAGA-K(Cy5)KDKPPR for tumor after normalization with healthy cerebellum or healthy brain.

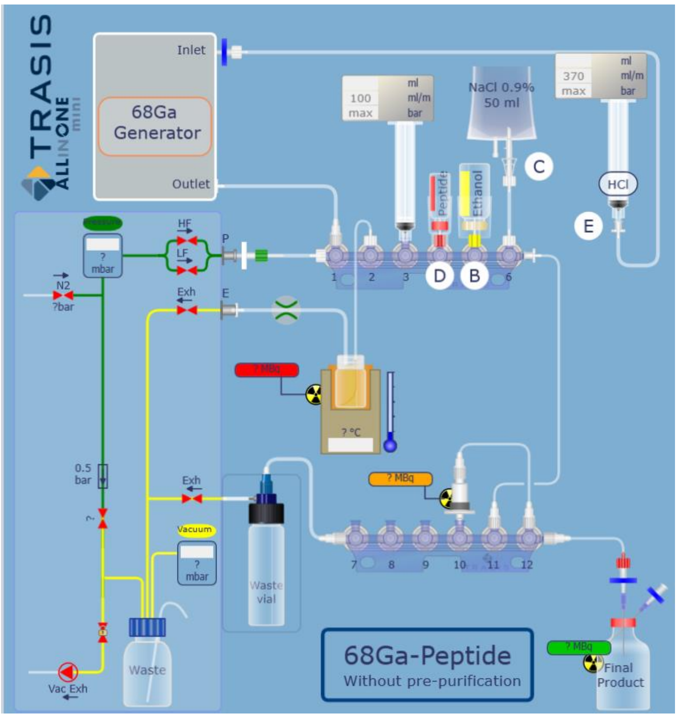

Figure S11. Graphical representation of single-used kit for automated radiosynthesis of [^{68}Ga]Ga-NODAGA-K(Cy5)DKPPR on mAIO (Trasis).

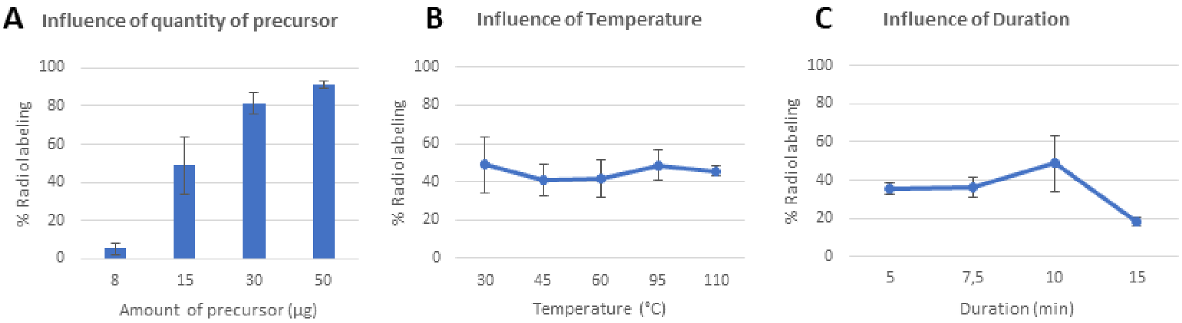

Figure S12. Result of radiochemical optimization for [^{68}Ga]Ga-NODAGA-K(Cy5)DKPPR synthesis. **A**: Influence of amount of precursor was evaluated in adding various quantity of precursor NODAGA-K(Cy5)DKPPR (8,15, 30, 50 µL) from a stock solution of 1 mg.mL^{-1} at 30°C for 10 min. (n ≥ 3). **B**: Influence of temperature was evaluated with 15 µg of precursor NODAGA-K(Cy5)DKPPR diluted in 0.8 M AcONa 1 mL for 10 min (n ≥ 3). **C**: Influence of reaction time was evaluated with 15 µg of precursor NODAGA-K(Cy5)DKPPR diluted in 0.8 M AcONa 1 mL at 30 °C (n ≥ 3).
